# Supplementary figures and images for: MAVS regulates the quality of the antibody response to West-Nile Virus
Source: PLoS Pathog. 2020 Oct 26;16(10):e1009009. doi: 10.1371/journal.ppat.1009009 (PMC7644103; doi:10.1371/journal.ppat.1009009)

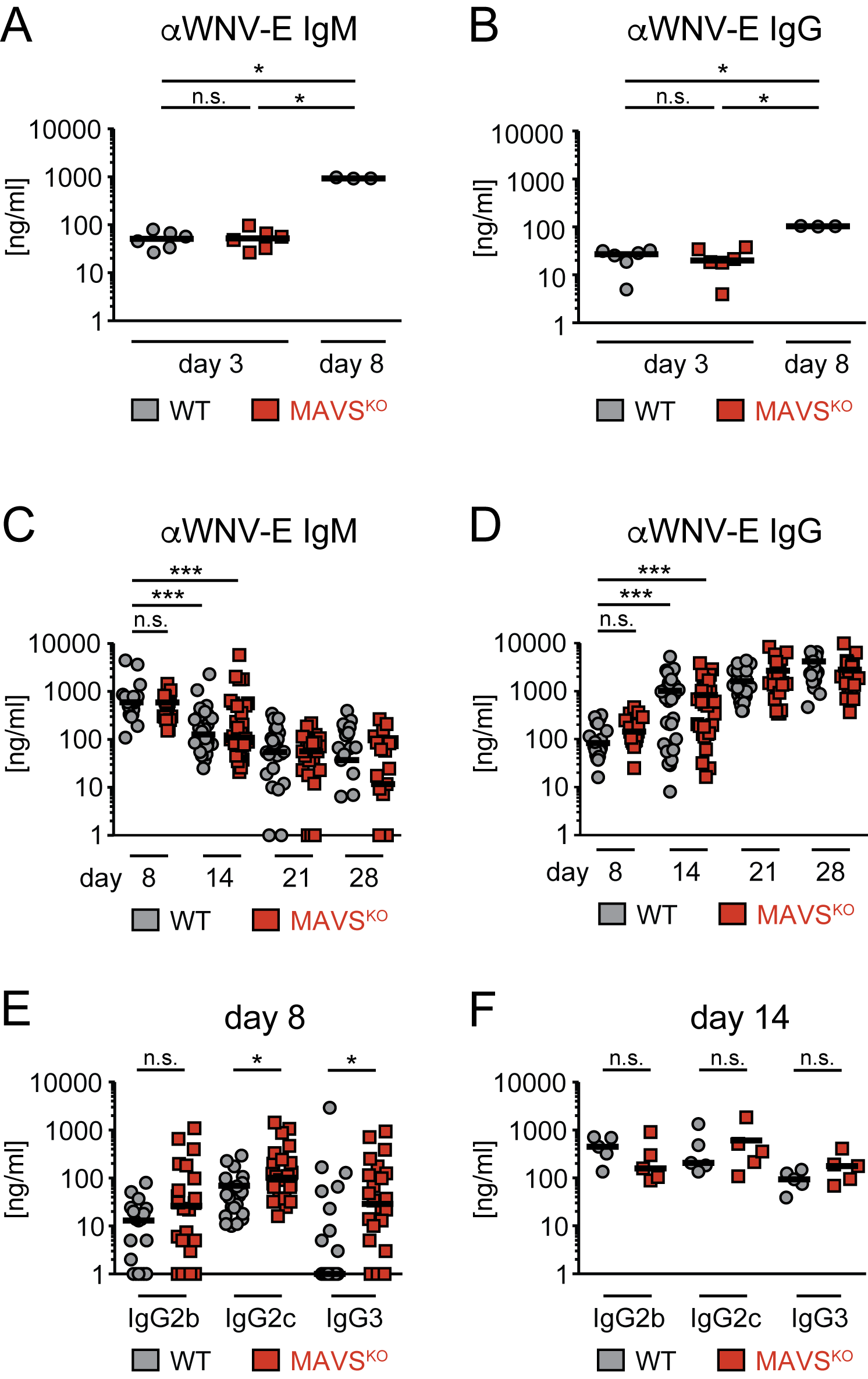

Supplement: S1 Fig — (A, B) WNV-E-specific IgM (A) and IgG (B) response on day 3 after infection with RWN (105 pfu/footpad) as measured by ELISA. Wild-type sera from day 8 were used as positive control. (C, D) WNV-E-specific IgM (C) and IgG (D) response over the course of 28 days after infection with RWN (105 pfu/footpad) as measured by ELISA. (E, F) WNV-E-specific IgG2b, IgG2c, and IgG3 titers on day 8 (E) and day 14 (F) post infection. Each dot represents one mouse, the lines represent the median. Shown are the combined data of 2–5 independent experiments. *, p <0.05; **; p < 0.005; ***; p < 0.0005; n.s., not significant; Mann-Whitney test. (TIF) [file ppat.1009009.s001.tif]

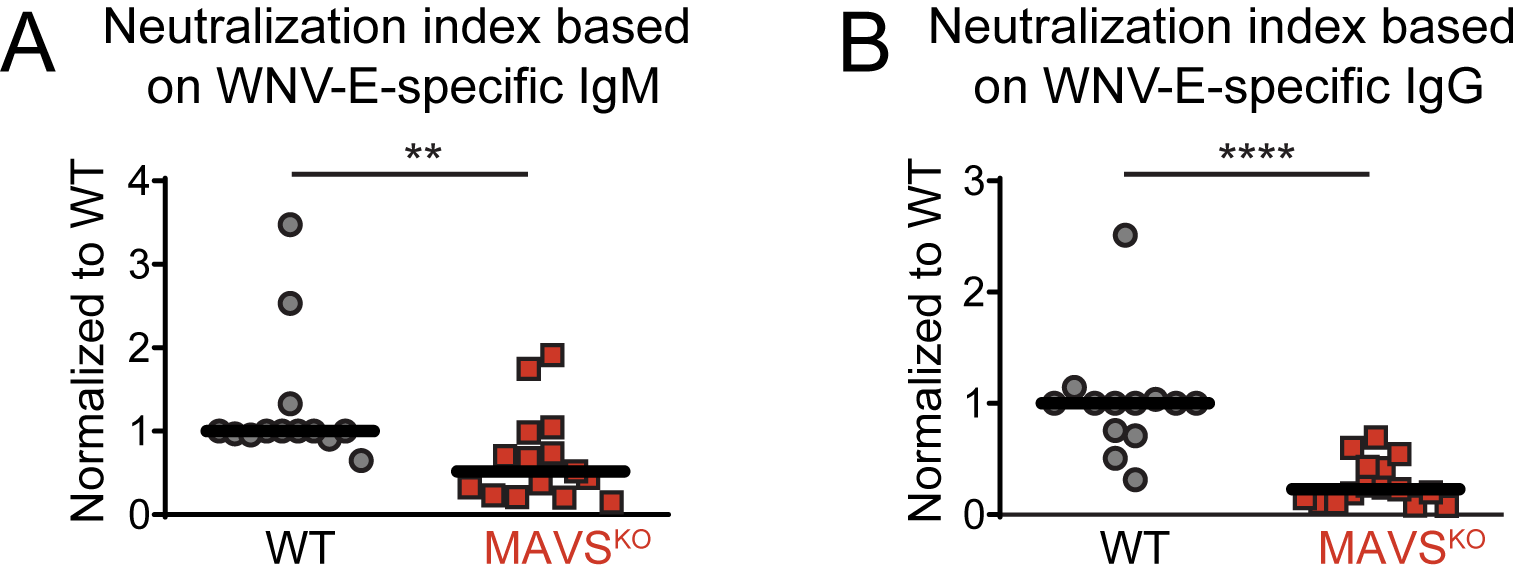

Supplement: S2 Fig — (A, B) Neutralization index calculated based on serum-specific levels of WNV-ENV-specific IgM (A) or IgG (B) titers on day 8 after infection. The data were normalized across multiple experiments to WT mice. Each dot represents one mouse, the lines represent the median. **, p <0.005; ****; p < 0.00005; n.s., not significant; Mann-Whitney test. (TIF) [file ppat.1009009.s002.tif]

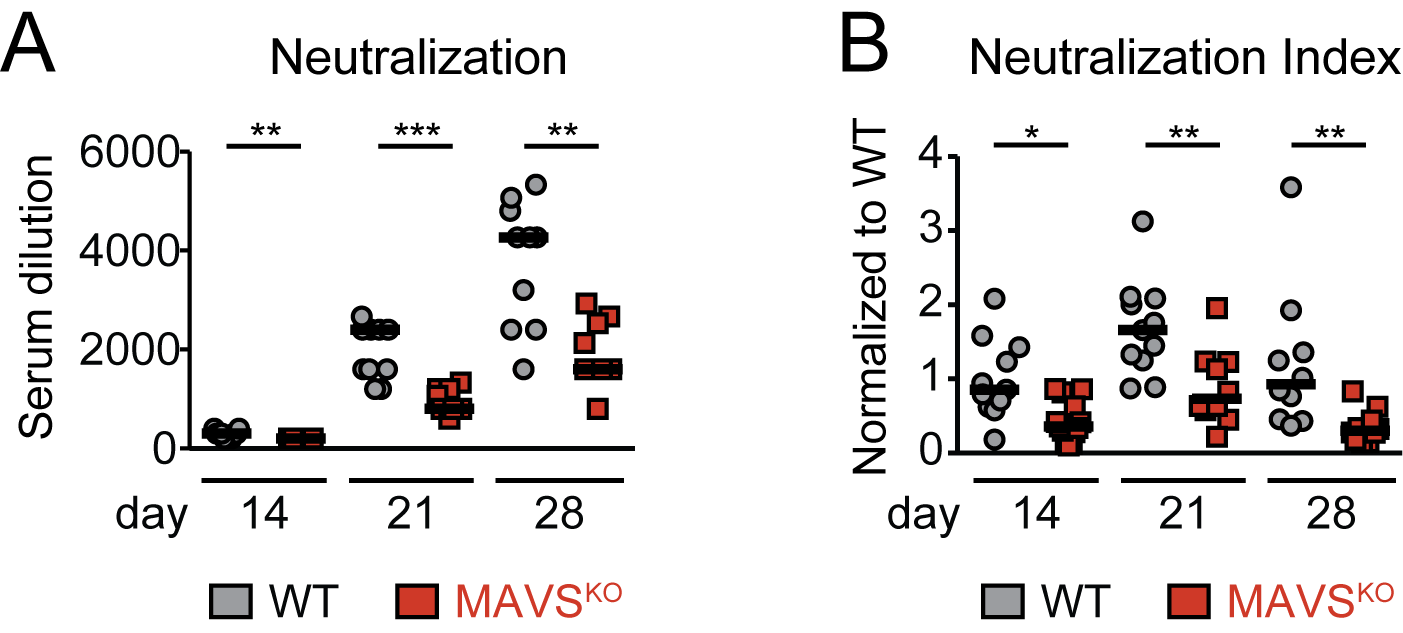

Supplement: S3 Fig — (A) Virus neutralization by sera of infected MAVSKO and MAVSWT mice. RWN was incubated with serial dilutions of sera prior to infection of Vero cells in vitro. The number of infected cells was determined two days later by staining with an anti-WNV-E antibody. The reduction of infected cells by 90% was scored (PRNT90). (B) Neutralization index reflecting virus neutralization relative to the total amount of WNV-E-specific antibodies in MAVSKO and MAVSWT mice. The index was calculated by dividing the dilution factor (PRNT90) of each mouse by the total amount of WNV-E-specific IgM and IgG of the same mouse. The data were normalized to the average of sera from MAVSWT mice on day 14. Each dot represents one mouse, the lines represent the median. Shown are the combined data of two independent experiments. *, p <0.05; **, p <0.005; ***; p < 0.0005; Mann-Whitney test. (TIF) [file ppat.1009009.s003.tif]

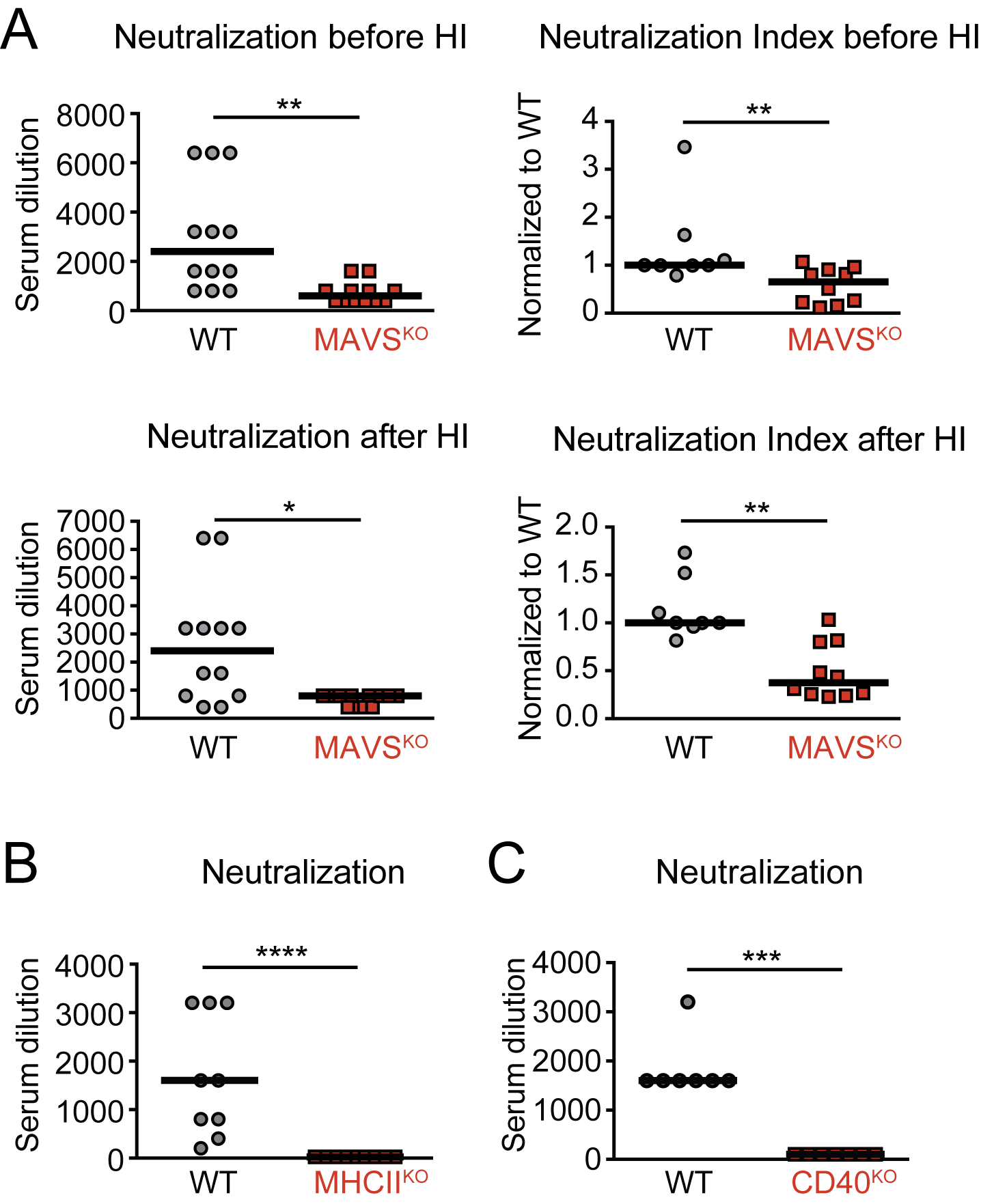

Supplement: S4 Fig — (A) Virus neutralization before and after heat-inactivation (HI) to exclude complement-mediated effects. Shown are the dilution factors that resulted in a 90% reduction of infection of target cells with RWN in vitro (PRNT90) and the neutralization index that accounts for the anti-WNV-E IgM and IgG titers in each mouse. (B) MHCIIKO and (C) CD40KO mice as well as WT controls were infected with 105 Pfu of RWN in the footpads. Serum was collected 8 days later to measure the dilution factor. Shown are the combined data of two independent experiments. Each dot is one mouse. *, p < 0.05; **, p < 0.005; ***, p < 0.0005; ****, p < 0.00005; Mann-Whitney test. (TIF) [file ppat.1009009.s004.tif]

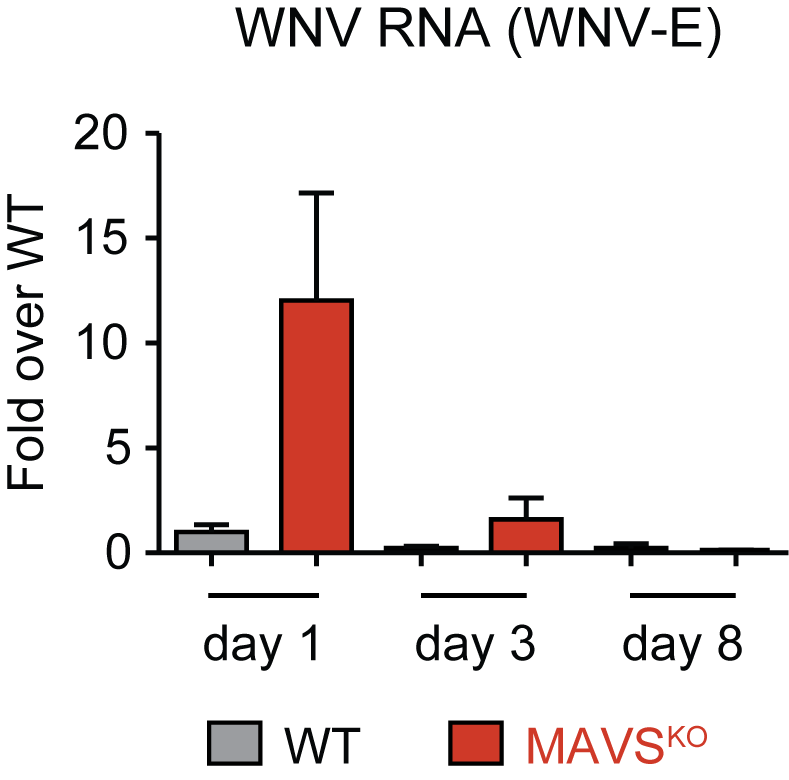

Supplement: S5 Fig — Viral RNA levels in the dLNs over the course of 8 days post infection with RWN (105 pfu/footpad) as measured by qPCR using primer pairs located in the WNV-E gene of the viral genome. Data were normalized to the RNA level of RWN-infected MAVSWT mice on day 1 post infection. (TIF) [file ppat.1009009.s005.tif]

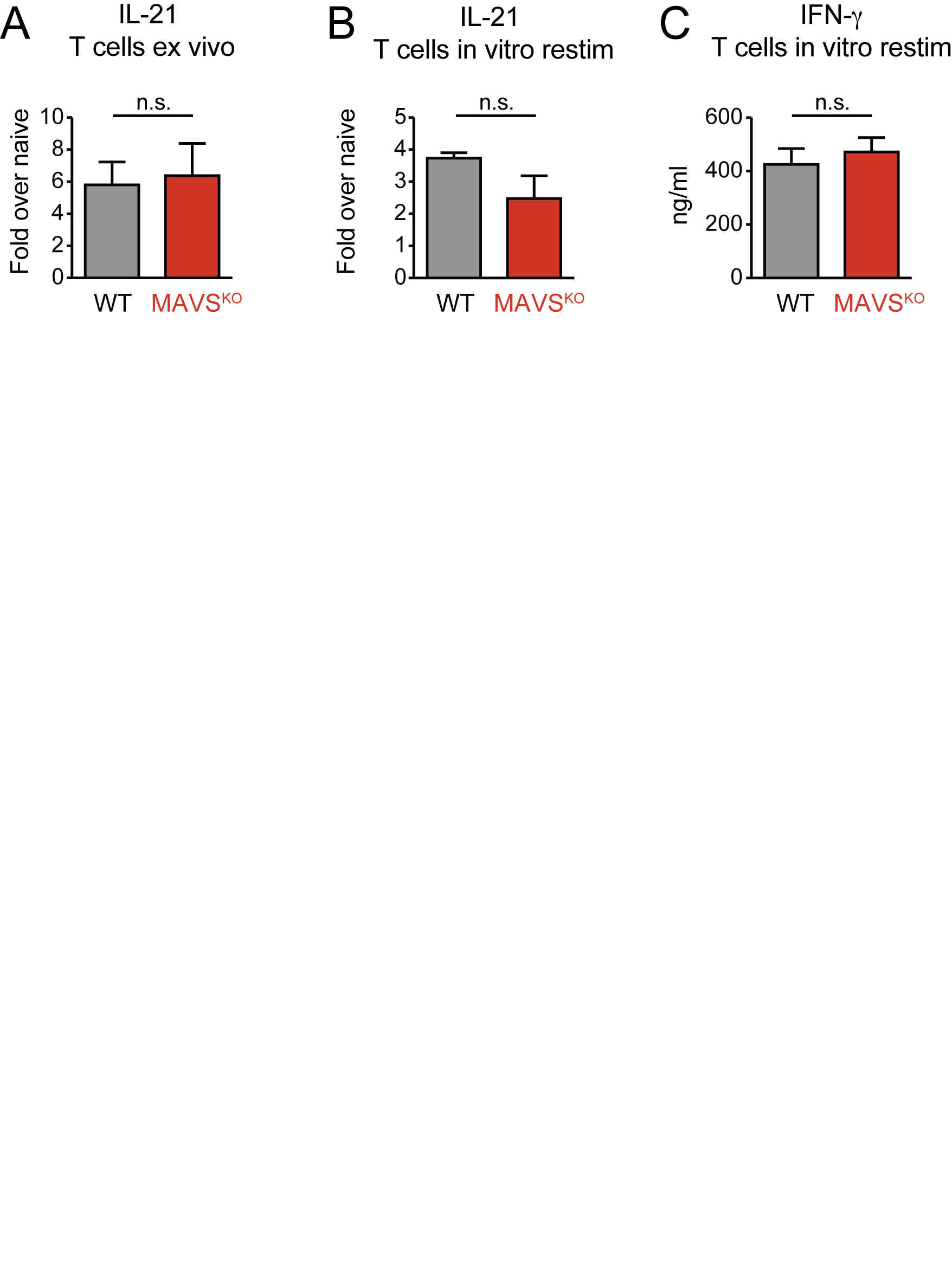

Supplement: S6 Fig — (A) Expression of IL-21 in CD4+ T cells isolated on day 8 from the dLNs of MAVSKO mice and MAVSWT controls as measured by qPCR. Shown is the expression over that of naïve MAVSWT mice. Expression of GAPDH was used to normalize the samples. (B) Expression of IL-21 in isolated CD4+ T cells from the dLNs three days after restimulation with E641 in the presence of naïve splenocytes as antigen-presenting cells as measured by qPCR. (C) Production of IFN-γ by isolated CD4+ T cells from the dLNs following the restimulation with E641 as measured by ELISA. Shown are the combined data of three independent experiments representing a total 6–10 mice per genotype. n. s., not significant; Mann-Whitney test. (TIF) [file ppat.1009009.s006.tif]

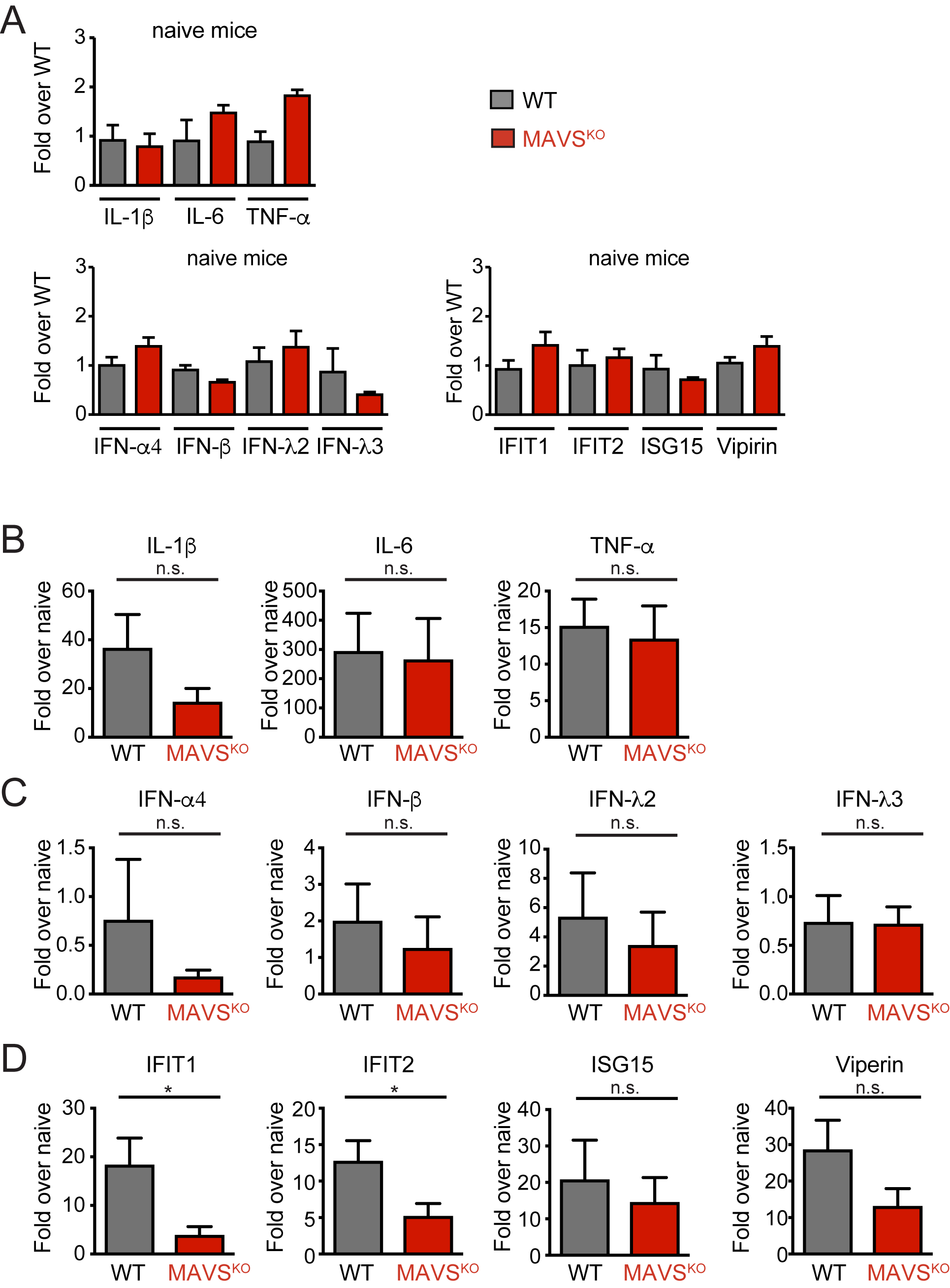

Supplement: S7 Fig — (A) Expression of indicated cytokines, IFNs, and ISGs in naïve MAVSKO and WT mice. Expression of GAPDH was used to normalize the samples. Shown are the combined data of 2 experiments using 3–4 mice/genotype. Differences between MAVSKO and WT mice were not significant. (B) Expression of IL-1β, IL-6, and TNF-α mRNA RWN-infected MAVSKO and MAVSWT mice on day 2 post infection. (C) Expression of type I and type III IFNs mRNA in RWN-infected MAVSKO and MAVSWT mice on day 2 post infection. (C) Expression of representative ISGs in RWN-infected MAVSKO and MAVSWT mice on day 2 post infection. (B-D) mRNA was isolated from whole dLN cells of mice 48 hours after infection with 105 Pfu RWN/footpad and measured by qPCR. Shown is the expression over that of dLNs from naïve WT mice. Expression of GAPDH was used to normalize the samples. Shown are the combined data of 4 experiments using 8–12 mice/genotype **, p <0.005; ***, p < 0.0005; Mann-Whitney test. (TIF) [file ppat.1009009.s007.tif]

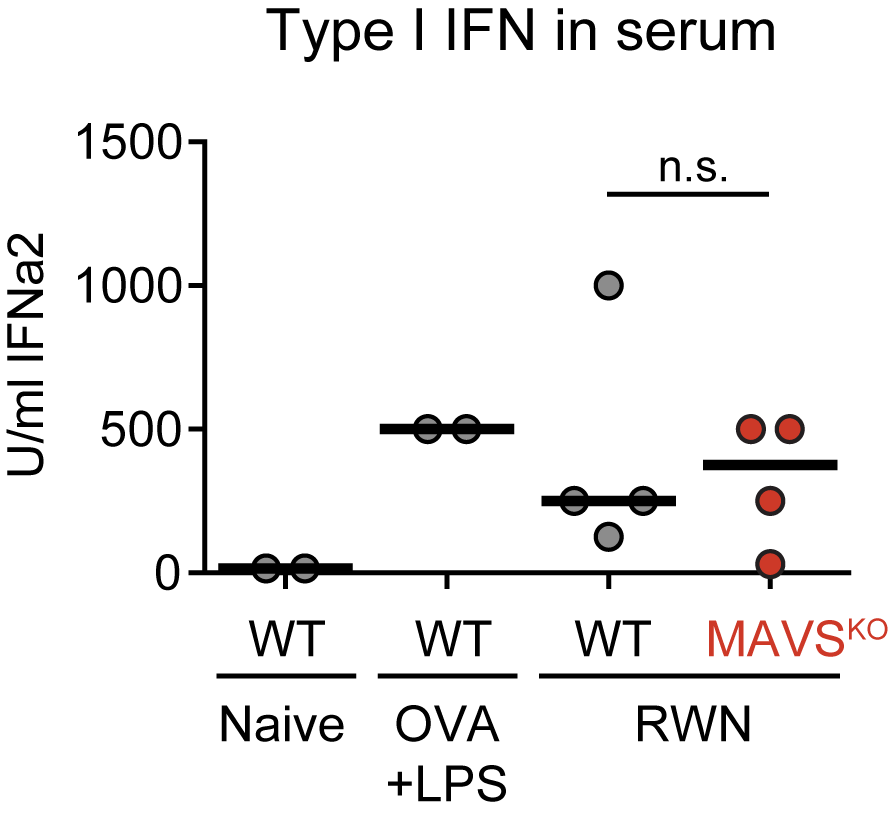

Supplement: S8 Fig — Serially diluted serum samples from day 1 of RWN-infected mice were used to protect L929 cells from the cytopathic effects (CPE) of vesicular stomatitis virus (VSV) in vitro. Samples from mice immunized with OVA + LPS were used as positive controls. All samples were compared to samples treated with increasing doses of recombinant IFN-α2 as standards. Shown are the combined data from two experiments. Each dot represents one mouse. n. s., not significant; Mann-Whitney test. (TIF) [file ppat.1009009.s008.tif]

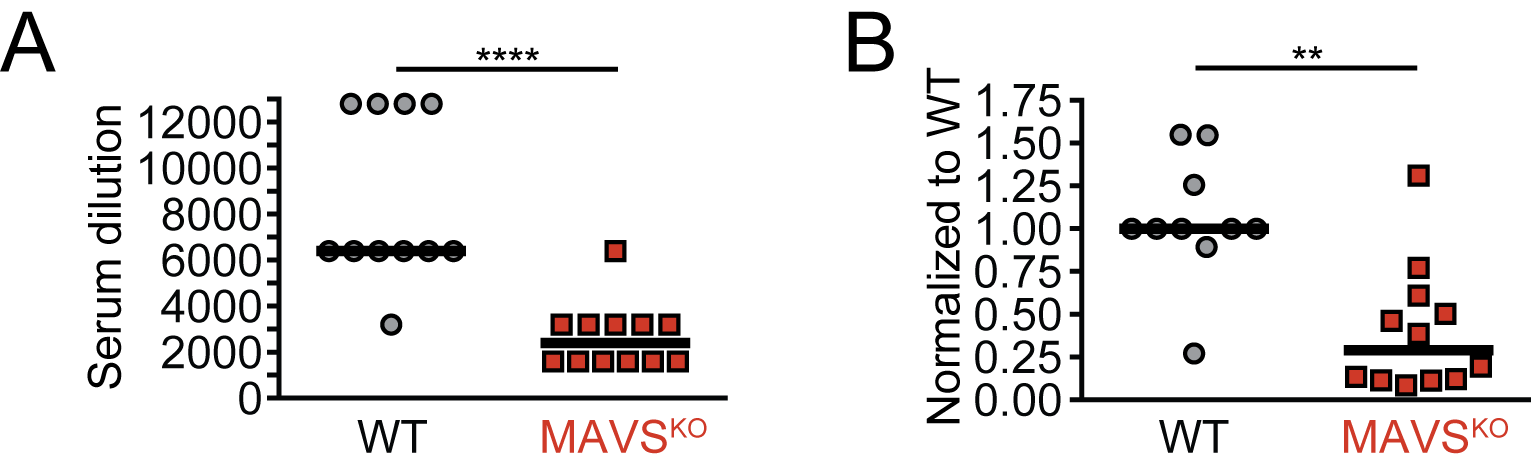

Supplement: S9 Fig — (A) Virus neutralization with sera from MAVSKO and MAVSWT mice. (B) Neutralization index for the same mice based on the DIII-specific IgM and IgG titers (Dilution factor divided by the total amount of DIII-specific IgM and IgG). Each dot represents one mouse, the line is the median. Shown are the combined data of two experiments. **, p <0.005; ****, p <0.00005; Mann-Whitney test. (TIF) [file ppat.1009009.s009.tif]
